# IOEASMA: an integrated clinical and educational pathway for managing asthma in children and adolescents

**DOI:** 10.1186/s13052-017-0374-8

**Published:** 2017-06-24

**Authors:** Sebastiano Guarnaccia, Gaia Pecorelli, Marina Bianchi, Massimo Cartabia, Gianluigi Casadei, Ada Pluda, Cristina Quecchia, Valeria Gretter, Maurizio Bonati

**Affiliations:** 1 “Centro Io e l’Asma”, Ospedale dei Bambini, ASST Spedali Civili di Brescia, Brescia, Italy; 2 Laboratorio per la Salute Materno Infantile. Istituto di Ricerche Farmacologiche Mario Negri, Milan, Italy; 3 CESAV, Istituto di Ricerche Farmacologiche Mario Negri, Milan, Italy

## Abstract

**Background:**

Due to the lack of real life clinical and educational studies, “Io e l’Asma” Centre performed this implementation research (IR).

Evaluate long-term effectiveness on bronchial asthma control of an integrated clinical and educational pathway for asthmatic children and adolescents.

**Methods:**

An observational retrospective pre-post intervention IR study was conducted among 262 children with asthma, ages 6-15 yrs. The intervention protocol included three clinical visits 8 weeks apart; an educational course at visit 1, post intervention consisted in two follow-up visits 6 months apart. The primary outcome was to verify the percentage of children who achieved bronchial asthma control at each visit. Secondary outcomes were based on daily therapy modulation, hospital admissions and the number of school days missed. An economic assessment was also included.

**Results:**

Two hundred sixty two children with bronchial asthma completed the pathway and were included in the analysis. The percentage of children who obtained disease control increased from 44% at visit 1 to 79% at visit 3 and at 1-year follow-up was 83%. Hospital admissions represent 11% of children: 8% before the intervention, 2% during the intervention, and 1% before and during the intervention; no hospitalizations related to bronchial asthma exacerbations were reported during the 2 follow-up visits.

**Conclusions:**

The therapeutic-educational pathway was adapted according to the international guidelines and the primary performance indicators. Our findings confirmed that the clinical plus educational approach, shared between specialists and family physicians, is an effective template for asthma management. These findings also demonstrated a strong economic advantage.

## Background

Bronchial asthma is the most frequent childhood chronic disease and can significantly undermine quality of life in children and their families [1]. Despite the availability of international guidelines on the management of asthma they are not fully followed by physicians and stakeholders [2, 3], and hence asthma remains under-diagnosed and under-treated [4–7]. International guidelines suggest that physicians and other care providers should educate patients and families, and that education concerning self-management of the disease should be reinforced at follow-up as part of the therapy [8–12]. Several studies report that the collaboration between the specialist and the family doctor can bring improved management of the disease including modulating daily therapy and preventing exacerbations [7, 13]. Moreover, ongoing monitoring of therapy is one of the most effective ways to avoid exacerbations and to maintain the control of disease [14]. Adherence to guidelines should lead to better management of asthma, improved disease control and to savings resulting from reduced emergency department (ED) admissions and/or hospitalizations.

A Canadian study [15] and GINA guidelines report evidence on key factors leading to optimal management of asthma: asthma education from a certified asthma educator, pulmonary function monitoring, and asthma control monitoring. In our study, we have adopted these key factors in order to monitor asthma control by developing a therapeutic-educational pathway, which includes three clinical visits at 8 weeks interval and two follow-up visits at 6-month intervals. After the first visit the patient and their parents receive an educational course. The “IOEASMA” pathway is based on a very strong collaboration of integrated care between clinicians and therapeutic educators. Achieving control through the pathway shared between the specialist and the primary care doctor means focusing on both impairment and risk [16].

## Methods

### Study population

Three hundred sixty two children and adolescents, 6–15 years of age, with bronchial asthma diagnosis, were recruited consecutively from the “Io e l’Asma” Center, Brescia, Italy; initially addressed by family pediatricians and primary care physicians [17], and subsequently structured as part of an integrated pathway lead by a multidisciplinary care team. This study was reviewed and approved by the Research Ethics Committee, ASST Spedali Civili, Brescia, Italy n° 2046, June 15, 2015. A written informed consent was obtained.

### Study design

This real-life implementation study has adopted a pre- to post-intervention design. The therapeutic-educational pathway includes three visits with an 8-week interval. Following the first visit an individual asthma education course was offered to all enrolled patients (Table 1). The three clinical visits were followed by two visits six months apart. The first assessment included family history, past medical history (PMH) and history of present illness (HPI). During the pathway prick-test and spirometry were performed. Once the control of the disease was evaluated, daily therapy was introduced or modified. At the second, third and two follow-up visits, symptoms were monitored, disease control was evaluated and daily therapy was adjusted as directed by GINA guidelines.

**Table 1:** Individual educational course integrated in the “IOEASMA” pathway

Individual educational course
Addressed to: children, parents, grandparents, other caregivers
When/ Where: after 1^st^ visit / in dedicated setting
Duration: 30 min
Aim
• Improve adherence to therapeutic pathway
• Become proactive in the every day management
Contents
• Prevention measures
• Early recognition of symptoms with action plan
• Appropriate use of drugs
• Encourage healthy life styles (sports, play)
• Keep diary for symptoms/ monitoring

### Outcome measures

- *Assessment of asthma control*
  According to GINA guidelines, the assessment of asthma control was evaluated during the 8 weeks preceding each clinical visit. Assessment criteria were based on the following GINA elements: daytime symptoms, limitation of activities, nocturnal symptoms/awakenings, need of reliever/rescue therapy, lung function (PEF or FEV1) and exacerbations.
- *Long term improvement (%) in asthma control*
  The percentage of children/adolescents who benefited from the intervention was calculated between the pre-intervention period (8 weeks observational period prior to the first visit), the intervention period (three visits at 8-week intervals) and the post-intervention period (two follow-up visits at 6-month intervals).
- *Hospitalization*
  Admissions for asthma exacerbations include hospitalization during the 8 weeks preceding each of the three visits and during the two follow-up visits.
- *Missed school days due to asthma*
  Missing school days was considered an indicator if the child was absent for at least one day from school due to asthma symptoms. The period taken into consideration was the 8 weeks preceding each of the three visits and the two follow-up visits.
- *Post-intervention dropouts*
  The number of dropout children was calculated during the post-intervention period, respectively at six months and one year.

### Statistical analysis

Data was collected in a Microsoft Access database. The comparison of the percentage of controlled children before and after the pathway has been calculated using the Chi-square test. To avoid possible differences due to the seasonality, the Mantel Haenszel Chi-square test was carried out in order to stratify children three months before and after the pathway. The independent variables considered were: gender; ethnicity (caucasian/non-caucasian); family medical history, parents’ smoking habits, assessment of asthma control at visit 1 (well-controlled/ partially controlled/ uncontrolled); comorbidities (conjunctivitis, atopic dermatitis, rhinitis), hospitalization, modulation of daily drugs (none, diminished, suspended, introduced, increased); seasonality (trimester in which the pathway has started). The stepwise regression model was utilized at the 0.10 level.

### Economic assessment (EA)

The EA was carried out following the Italian National Health Service (NHS) perspective. The following direct health costs were considered: i) healthcare services, including the three ambulatory visits, educational course, prick test, and spirometry; ii) hospitalization, and iii) reimbursed drugs.

Selected costs were estimated referring to the following sources: a) tariffs reimbursed by Lombardy region, where the center is located; and ii) hospitalization reimbursed cost related to DRG code M098- bronchitis or asthma in patients aged <18 years. Finally, the price-to-public of each prescribed drug was obtained from Farmadati (http://www.farmadati.it/) and the daily cost has been calculated.

## Results

**Fig. 1:**
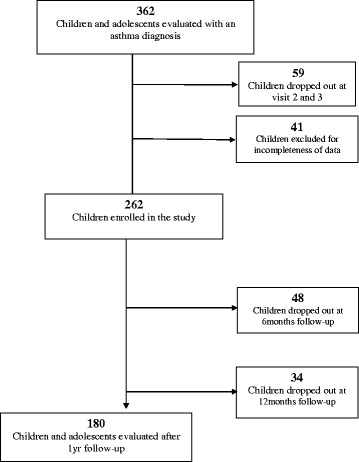
Subjects enrolled and evaluated in the study

Of the 362 children, fifty-nine voluntarily dropped out during the intervention and forty-one were excluded due to incomplete data in the medical records. 262 children/adolescents completed the pathway, of these, forty-eight children (18.3%) dropped out at the first follow-up visit and thirty-four (15.9%) at the second follow-up visit (Fig. 1).

The characteristics of the pediatric population in the “IOEASMA” pathway (age, gender ratio, ethnicity, primary physician, smoking habits, and co-morbidities) are represented in Table 2.

**Table 2:** Characteristics of children of “IOEASMA” pathway at visit 3, at 6 months and 12 months follow-up

	3rd visit (*n* = 262)	6 months follow-up (*n* = 214)	12 months follow-up (*n* = 180)
	N (%)		
Age (years)			
Mean; SD	9.5; 2.9	10.2; 3.1	10.6; 3.5
Gender			
Boys	169 (64.5)	139 (65.0)	115 (63.9)
Girls	93 (35.5)	74 (34.6)	65 (36.1)
Ethnicity			
Caucasian	225 (85.9)	185 (86.4)	156 (86.7)
Non Caucasian	37 (14.1)	29 (13.5)	24 (13.3)
Physician			
Paediatrician	156 (59.5)	137 (64.0)	113 (62.8)
General Practitioner	106 (40.5)	77 (36.0)	67 (37.2)
Smoking Habits			
No	174 (66.4)	141 (65.9)	115 (63.9)
Yes	88 (34.6)	73 (34.1)	65 (36.1)
Atopic dermatitis symptoms	41 (15.6)	38 (17.8)	35 (19.4)
Rhinitis symptoms	6 (2.3)	5 (2.3)	4 (2.2)
Bronchodilator use	205 (78.2)	173 (80.8)	147 (81.7)
Rhinitis Therapies	34 (13.0)	30 (14.0)	20 (11.1)

### Dropouts during follow-up visits (one year)

As we mentioned, forty-eight (18.3%) and thirty-four (15.9%) subjects dropped out, respectively, at the six months and one-year follow-up visits. The percentages of well -controlled patients who dropped out at six months, with and without a daily therapy were, respectively, 4.2% and 14.1%; and 3.7% and 12.1% at one year follow-up.

### Changes in asthma control

**Fig. 2:**
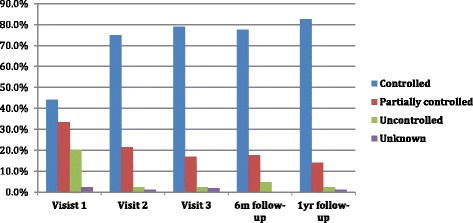
Asthma control during the intervention period and at 6 months and 1 year follow-up
- *Variation of level of asthma control during intervention period (three visits)*
  44% of patients have modified levels of asthma control during the intervention: 23% shifted from partly controlled to well-controlled; 17% went from uncontrolled to well-controlled; and 4% from uncontrolled to partially controlled.
- *Daily therapy modification after post intervention period (one year)*
  The utilization of drugs changed as follows: fluticasone decreased from 49% to 19.4%, montelukast and salmeterol/fluticasone decreased from 4% to 3% both.
- *Modulation of daily therapy during intervention period (three visits)*
  After three visits, 42% of patients started with the daily therapy; 25% did not receive any; 15% continued with the same therapy; 11% required a modulation; 9% suspended daily therapy.
- *Changes in inhaled steroid dosages therapy during the intervention period (three visits)*
  The percentage of children/adolescents receiving daily therapy increased from 30% to 53%. Comparing the therapy between the third and first visit, 30% of children started a daily therapy or introduced a second drug; 48% did not receive any steroid treatment; while 19% maintained the treatment established by the primary care physician at the first visit; 17% have suspended the treatment. It is important to mention that the modulation of drugs used, showed that there was an increase in patients receiving fluticasone from 16% to 49%. At the end of the intervention (third visit), among the 49% of patients treated with fluticasone, 30% of children were treated with 100mcg/day and 19% ranged between 100-250mcg/day. The percentage of children/adolescents who properly used the inhaler device was 75% at the first visit and 100% at the third.

- *Percentage of children and adolescents with well-controlled asthma*
  The percentage of children and adolescents with well-controlled asthma increased from visit 1 (44%) to visit 3 (79%, χ2 = 66.8; *p* < 0.0001). Well-controlled asthma was achieved in 79% of children at six months and increased at 83% after one year (Fig. 2). At visit 3, 32% of children no longer required daily therapy. However, 31% of the percentage (79%) of well-controlled patients needed introduction or increase of daily therapy.

### Hospitalization for asthma

11% of children were hospitalized: 8% before the intervention 2% during the intervention and 1% before and during intervention (χ^2^ = 15.4; *p* = 0.0001). No subjects underwent hospitalization at one year follow-up.

### Missed school days because of asthma

20% of children missed at least one day of school because of asthma: 14% before the intervention only, 4% during the intervention only, and 2% before and during the intervention (comparison pre-post: χ^2^ = 11.5; *p* = 0.0007). 3% and 2% of children have missed school days, respectively at the six months and one year follow-up visits.

### Long-term benefits

The results of the analysis indicated that variables associated with a long-term benefit were the following: presence of co-morbidities, need for daily therapy at the first visit, modulation of anti-asthmatic medications prior to the intervention (Table 3).

**Table 3:** Chi-square test for independence (α = 0.10)

Variable	d.f.	chi-square	*p*-value
Gender	1	0.74	0.3900
Ethnicity	1	0.83	0.3600
Familial atopic anamnesis	1	0.64	0.4200
Parent smokers	1	0.86	0.3500
Diagnosis (at visit 1)	1	8.50	0.0035*
Associated allergic pathologies (at visit 1)	1	78.90	<0.0001*
Hospitalization	2	3.48	0.1800
Anti-asthma drug use	2	48.01	<0.0001*

*statistically significant at *P* ≤ 0.05

The logistic regression analysis indicated that the variables associated with a clinical benefit were diagnosis of persistent asthma at the first visit (OR = 21.7; IC 90% = 11.3–41.6) and at least one hospitalization before accessing the pathway (OR = 3.28; IC 90% = 1.1–9.5).

### Economic assessment

According to the National Health System (NHS) perspectives the estimated total cost of the intervention, including the three evaluations (three clinical visits, prick test, spirometry and educational course), was €269.27 per patient. The median daily drug cost was €0.26 per patient (IQR = 0.26). Fluticasone at lower dosage (100 microgram/die) was the most prescribed drug, accounting for 87% of the total days of treatment and its mean cost (0.35 €/die) was the lowest among the prescribed drugs. The remaining most frequently prescribed drugs were: montelukast (€0.52; 6% of total days of therapy), and salmeterol/fluticasone (€0.69; 4% of total days of therapy). Formoterol/budesonide and budesonide accounted for the remaining 3%. Overall, the cost of pharmacological therapies in the post intervention period decreased by 48% compared to the pre intervention.

The cost of hospitalizations was not assessed because no admission related to asthma exacerbations was reported during the two 6-month follow-up visits.

## Discussion

Bronchial asthma is a chronic disease. The overriding goal of therapy is to obtain and maintain control over time. In this study, asthma control improved from 44% at baseline to 79% at visit 3; that control was maintained during the following six months and increased to 83% after one-year follow-up. In 38% of patients, asthma control was obtained and maintained without a daily pharmacological therapy. In most cases, a low dose of inhaled steroids such as fluticasone when combined with therapeutic education (i.e. prophylaxis measures, adherence to therapeutic educational plan, promptly use of beta2 agonist) consistently achieved positive asthma control. In five subjects (1.9%) during the intervention period and in one subject (0.6%) at one year follow-up it was necessary to utilize the combination of inhaled corticosteroids and long-acting beta2 agonist (ICS-LABA) in order to achieve asthma control. For comparison purposes, during 2005 in the entire Lombardy Region, 2% of subjects (6–17 years old) recently diagnosed with asthma received prescriptions of LABA alone and 23% received prescription of the combination ICS-LABA [18, 19]. The cost of drug therapies at the one-year follow-up decreased by 48% and the percentage of asthma control improved. Another recent study indicated that a minimal dose of ICS is effective in children with asthma and does not have an impact on growth over a one-year period; although parents and physicians remain concerned about the potential negative effect that ICS could still have on growth [20].

This IR effectiveness study demonstrates that well-managed care and strong patient-provider communication, which in turn leads to, improved family management of the asthma, permits patients to control their asthma with low-dose inhaled steroids. The highest percentage of drop-outs was among well-controlled patients without therapy, at six months and one year.

The family pediatrician/general practitioner should provide the education session during the clinical visit, which includes demonstration and assessment of child device technique [21], and review of specific signs and symptoms to guide daily therapy [22]. To this regards Vernacchio et al. [23] designed a new program for asthma quality improvement, developing practice-based registries of children 5 to 17 years of age with persistent asthma and helped physicians improve processes of asthma care through education, data feedback and sharing of best practices.

Unfortunately, the main limitation of this real life outpatient study is that it does not have a control group. On the other hand, the evaluation of a real life integrated approach such as “IOEASMA” therapeutic-educational pathway represents an innovative way for patient-centered care between primary physicians and specialists. These studies should be encouraged in the “real world” and more perspective should be given to identify the best approach to therapy for the individual child. In addition, we suggest examining the incorporation of a comprehensive health promotion intervention (i.e. smoking, nutrition, physical activity, psychosocial) into a clinical setting, by using a multidisciplinary health care approach and by building on the successes of other clinical asthma interventions [24].

Interpretation of results is subject to the study’s limitations. This was not a randomized trial. Study investigators were in many cases the treating physicians who provided their own assessments of control. Patients who dropped out may have been those less well managed and controlled, potentially biasing results. The cost assessment could not account for indirect costs. Nonetheless, IR effectiveness studies such as this one have the strong advantage of assessing treatment success in real world settings. IR research is particularly important because it creates a mechanism for assessing whether interventions tested in randomized controlled trials will disseminate into day-to-day practice [25, 26].

The therapeutic-educational pathway was adapted according to the international guidelines and the primary performance indicators. The short- and long-term results in every day practice confirm the importance of building a pathway where specialists, primary care physicians and stakeholders can coordinate care to deliver the most personalized asthma management.

## Conclusions

The evaluation of a real-life integrated approach such as “IOEASMA” therapeutic-educational pathway represents an innovative way of patient-centered care to interact between primary physicians and specialists.

These studies should be encouraged in the “real world” and more perspective should be given to identify the best approach to therapy for the individual child.

We suggest examining the incorporation of a comprehensive health promotion intervention (i.e. smoking, nutrition, physical activity, psychosocial) into a clinical setting, by using a multidisciplinary health care approach and by building successes of other clinical asthma interventions.

This implementation research is important because it creates a mechanism for assessing whether interventions tested in randomized controlled trials will disseminate into day-to-day practice.

The short- and long-term results in every day practice confirm the importance of building a pathway where specialists, primary care physicians and stakeholders can coordinate care to deliver the most personalized asthma management
